# USF1-mediated upregulation of lncRNA GAS6-AS2 facilitates osteosarcoma progression through miR-934/BCAT1 axis

**DOI:** 10.18632/aging.103015

**Published:** 2020-04-08

**Authors:** Guojun Wei, Tianwei Zhang, Zongguang Li, Naichun Yu, Xiang Xue, Daguo Zhou, Yongjie Chen, Linlin Zhang, Xiaoli Yao, Guangrong Ji

**Affiliations:** 1 Department of Orthopaedics, The Xiang’an Hospital of Xiamen University, School of Medicine, Xiamen University, Xiamen 361101, Fujian, China; 2 Department of Anesthesiology, The First Affiliated Hospital of Xiamen University, Xiamen 361003, Fujian, China; 3 Department of Gastroenterology, The Xiang’an Hospital of Xiamen University, School of Medicine, Xiamen University, Xiamen 361101, Fujian, China

**Keywords:** GAS6-AS2, USF1, miR-934, BCAT1, osteosarcoma

## Abstract

Long noncoding RNAs (lncRNAs) have been certified as important regulators in tumorigenesis. LncRNA GAS6-AS2 (GAS6-AS2) was a newly identified tumor-related lncRNA, and its dysregulation and oncogenic effects in melanoma and bladder cancer had been reported in previous studies. However, the expression pattern and potential function of GAS6-AS2 in osteosarcoma (OS) have not been investigated. In this study, we identified a novel OS-related lncRNA GAS6-AS2. We found that GAS6-AS2 was distinctly upregulated in both OS specimens and cell lines. Distinct up-regulation of GAS6-AS2 in OS was correlated with advanced clinical stages and shorter survivals. In addition, USF1 could directly bind to the GAS6-AS2 promoter and contribute to its overexpression. Furthermore, GAS6-AS2 knockdown caused tumor suppressive effects via reducing cellular proliferation, migration and invasion, and promoting OS cell apoptosis. Besides, GAS6-AS2 directly bound to miR-934 and downregulated its expression. Mechanistically, GAS6-AS2 positively regulated the expression of BCAT1 through sponging miR-934. Taken together, our data illustrated how GAS6-AS2 played an oncogenic role in OS and might offer a potential therapeutic target for treating OS.

## INTRODUCTION

Osteosarcoma (OS), characterized by an enormous potential in grade malignancy and metastasis, is the most common malignant bone neoplasm of the musculoskeletal system in children and adolescents [1, 2]. In China, the incidence of OS in recent years is raising and this tumor presents extensive clinicopathological behaviors [3]. Common clinical treatments of OS include chemotherapy and radiotherapy before and after surgery, which have improved the long-term survival for many patients [4, 5]. However, noticeable improvements have not been achieved in terms of survival of patients who had initial metastasis at diagnosis. In addition, tumor recurrence and drug resistance contribute to the unfavorable clinical outcome [6]. Up to date, the potential mechanism involved in the metastasis remains largely unclear. Therefore, for the improvements of therapeutic efficacy and sensitive biomarkers, it is urgently needed to develop a better understanding for pathogenesis of OS.

Long noncoding RNAs (lncRNAs) are defined as transcripts, each with a length of > 210 nucleotides and limited protein-coding abilities [7]. Growing studies reveal that lncRNAs play crucial roles in a wide array of biological cellular processes, such as gene expressions, cell growth, apoptosis [8, 9]. Given the regulator effects of lncRNAs in tumor-related genes, lncRNAs are supposed to exhibit their functional roles by functioning as either oncogenes or tumor suppressors in the progression of cancer [10, 11]. For instance, lncRNA LINC00339 was reported to facilitate the proliferation and invasion of lung cancer cells via regulating miR-145 [12]. LncRNA NKILA was shown to indict shorter overall survival of hepatocellular carcinoma patients and enhance the anti-tumor effects of baicalein in tumor cells through modulating NF-κB signaling [13]. In OS, lncRNA DANCR was reported to act as an oncogene via promoting the expression of AXL [14]. Nevertheless, a large number of uncharacteristic lncRNAs remained to be further functionally explored in OS. Hence, the identification of more tumor-associated lncRNAs is necessary for the study of the OS progression.

LncRNA GAS6-AS2 (GAS6-AS2), a newly discovered tumor-related lncRNA, was mapped to 13q34 with a transcript length of 12,361 bp. Up to date, the studies on the expression and function of GAS6-AS2 in tumors were limited. Previous findings had confirmed that GAS6-AS2 was highly expressed in both melanoma and bladder cancer tissues [15, 16]. In addition, its tumor-promotive roles in the above two tumors were also demonstrated in vitro assays. However, the expression and potential biological function of GAS6-AS2 in other tumors are unclear. In this study, we aimed to the clinical significance and possible function of GAS6-AS2 in controlling the progression of OS.

## RESULTS

### Highly expressed GAS6-AS2 in OS was associated with poor prognosis

**Figure 1:**
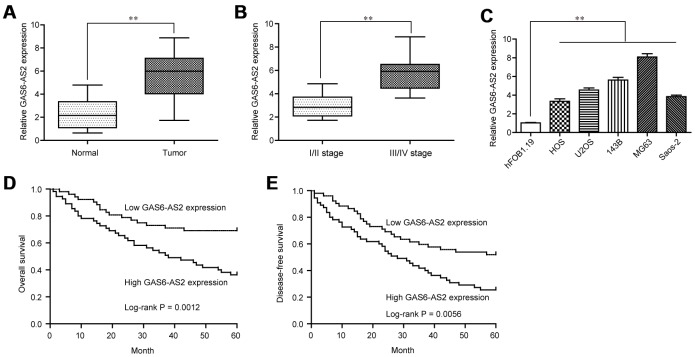
**GAS6-AS2 was highly expressed in OS tissues and associated with poor prognosis.** (**A**) The levels of GAS6-AS2 was detected in 157 pairs of OS tissues and matched normal tissues using qRT-PCR. (**B**) The expression trend of GAS6-AS2 in OS patients with different clinical stages. (**C**) RT-PCR assays for the expressions of GAS6-AS2 in five OS cell lines and normal bone cell. (**D**) Kaplan-Meier curves for overall survival in patients with OS. (**E**) Kaplan-Meier curves for disease-free survival in patients with OS. * P < 0.05, **P < 0.01.

To explore whether GAS6-AS2 was abnormally expressed in OS, we examined GAS6-AS2 expression using qPCR assays in 157 paired OS specimens and adjacent non-cancerous bone specimens, finding that GAS6-AS2 was distinctly highly expressed in OS specimens compared with matched normal tissues (*p* < 0.01, Figure 1A). In addition, higher expression of GAS6-AS2 was observed in OS patients with advanced stages (Figure 1B). Moreover, we also demonstrated that GAS6-AS2 was significantly upregulated in five OS cell lines compared to hFOB1.19 cells (Figure 1C). For further exploration of the effects of GAS6-AS2 on clinical progression of OS patients, we divided all the OS patients into a high expression group (n = 77) and a low expression group (n = 80) based on the median level of GAS6-AS2 in OS tissues. The results of chi-square test indicated that high expression of GAS6-AS2 was associated with clinical stage (*p* = 0.012) and distant metastasis (*p* = 0.021) (Table 1). Furthermore, whether GAS6-AS2 had a prognostic influence on the survival of OS patients was determined using Kaplan-Meier analysis. We found that higher GAS6-AS2 expressions were inversely associated with OS patients’ overall survival (p = 0.0012, Figure 1D) and disease-free survival (*p* = 0.0056, Figure 1E). Subsequently, we performed univariate assays and found several clinical variables which might be potential indicators of survivals (Tables 2 and 3). Furthermore, the results of multivariate assays indicated that GAS6-AS2 expression was an independent prognostic indicator of overall survival (HR=2.865, 95% CI: 1.147-4.042, *p* = 0.021, Table 2) and disease-free survival (HR=2.947, 95% CI: 1.157-4.653, *p* = 0.008) in patients with OS (Table 3). Overall, our findings indicated that up-regulation of GAS6-AS2 may be used to predict the clinical outcome of patients with OS.

**Table 1:** GAS6-AS2 expression and clinicopathologic features in osteosarcoma patients.

**Parameters**	**Category**	**No.**	**GAS6-AS21 level**		***p* value**
			**High**	**Low**	
Age(year)	<25	72	32	40	0.289
	≥25	85	45	40	
Gender	Male	91	43	48	0.598
	Female	66	34	32	
Tumor size	>8 cm	61	35	26	0.096
	≤8 cm	96	42	54	
Anatomical location	Tibia/femur	79	40	39	0.689
	Elsewhere	78	37	41	
Clinical stage	IIA	103	43	60	0.012
	IIB/III	54	34	20	
Distant metastasis	Absent	115	50	65	0.021
	Present	42	27	15	

**Table 2:** Univariate and multivariate analyses of overall survival in osteosarcoma patients.

**Factors**	**Univariate analyses**			**multivariate analyses**		
	**HR**	**95% CI**	***p***	**HR**	**95% CI**	***p***
Age(year)	1.662	0.774-2.371	0.365	-	-	-
Gender	1.528	0.528-2.091	0.298	-	-	-
Tumor size	1.772	0.894-2.347	0.118	-	-	-
Anatomical location	1.775	0.928-2.553	0.125	-	-	-
Clinical stage	3.546	1.427-5.217	0.004	3.018	1.178-4.572	0.014
Distant metastasis	3.287	1.397-4.896	0.007	2.896	1.219-4.262	0.016
GAS6-AS2 expression	3.156	1.316-4.672	0.009	2.865	1.147-4.042	0.021

**Table 3:** Univariate and multivariate analyses of disease-free survival in osteosarcoma patients.

**Factors**	**Univariate analyses**			**multivariate analyses**		
	**HR**	**95% CI**	***p***	**HR**	**95% CI**	***p***
Age(year)	1.358	0.872-1.895	0.218	-	-	-
Gender	1.472	0.649-2.174	0.143	-	-	-
Tumor size	1.785	0.895-2.442	0.089	-	-	-
Anatomical location	1.561	0.672-2.136	0.137	-	-	-
Clinical stage	3.319	1.327-5.427	0.004	3.018	1.218-4.775	0.009
Distant metastasis	3.275	1.218-4.882	0.006	2.886	1.058-4.274	0.014
GAS6-AS2 expression	3.175	1.327-5.018	0.003	2.947	1.157-4.653	0.008

### USF1 induced GAS6-AS2 expression via promoting its transcription.

**Figure 2:**
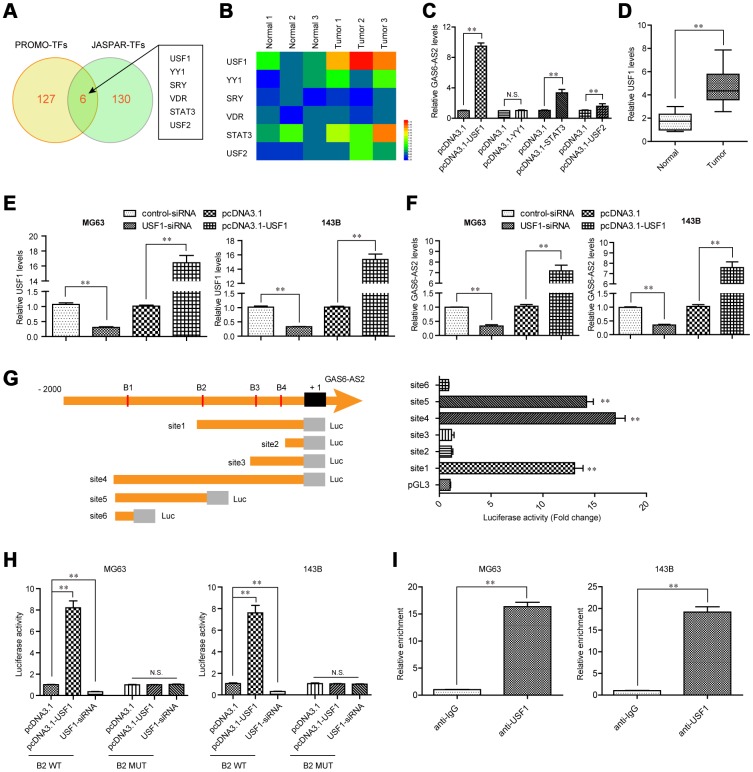
**USF1 was involved in GAS6-AS2 aberrant expression.** (**A**) Intersection of “PROMO” and “JASPAR” algorithms predicted binding transcriptional factors (TFs) in GAS6-AS2 promoter. (**B**) Real-time PCR detected the expression of TFs in randomly selected three paired OS tumor specimens and normal tissues. (**C**) qPCR determined the levels of USF1, YY1, STAT3 and USF2. (**D**) qPCR detected the levels of USF1 in 157 OS samples. (**E**) qPCR examined USF1 levels in MG63 and 143B cells after various treatment. (**F**) Real-time assessed GAS6-AS2 levels in MG63 and 143B cells after various treatment. (**G**) Left: schematic representation of constructs for GAS6-AS2 promoter luciferase reporter. B1-B4: “JASPAR” predicted binding sequences of USF1 in GAS6-AS2 promoter. Luc: luciferase. Right: luciferase activities of 6 truncated constructs (site1-6) in HEK-293 T cells. (**H**) Luciferase activity detection. B2 WT: luciferase reporter containing wild-type “JASPAR” predicted binding sequence 2 of USF1. B2 MUT: luciferase reporter containing mutant-type “JASPAR” predicted binding sequence 2 of USF1. (**I**) ChIP assays. * P < 0.05, **P < 0.01.

Since the above results revealed that GAS6-AS2 was up-regulated in OS and correlated with poor prognosis, we next sought to discover the molecular mechanisms which contributed to GAS6-AS2 high expression in OS. Emerging evidences had presented that transcriptional factors (TFs) might be involved in inducing lncRNAs expression via binding to their promoters [17, 18]. Therefore, we hypothesized that GAS6-AS2 high expression in OS was due to specific TF activation. To clarify that, we first employed “PROMO” and “JASPAR” algorithms to predict which TFs were able to binding to the promoter of GAS6-AS2. As the results presented in Figure 2A, there were six TFs in both algorithms predicting results. Hence, we next randomly selected three paired OS tumor specimens and normal tissues to examine the expressing levels of the predicted six TFs. The data demonstrated that only USF1, YY1, STAT3 and USF2 were up-regulated in the three OS tumor samples when compared with paired normal specimens (Figure 2B). Consequently, we constructed overexpressing plasmids of these four TFs and respectively transfected into OS cells. The data from qPCR analyses revealed that only USF1, STAT3 and USF2 were capable to stimulate GAS6-AS2 expression, and USF1 could increase remarkably higher levels of GAS6-AS2 than that of STAT3 and USF2 (Figure 2C). Therefore, we next focused on USF1 and aimed to prove that USF1 could lead to increased GAS6-AS2 expression via binding to its promoter. Indeed, qPCR analyses confirmed that USF1 was up-regulated in 157 OS specimens (Figure 2D). We next transfected siRNAs targeting USF1 (USF1-siRNA) or USF1 overexpressing plasmids into OS cells, and applied qPCR analyses to examine the expression of USF1. We found that USF1 was significantly knocked down by USF1-siRNA and pcDNA3.1-USF1 could markedly increase USF1 expression (Figure 2E). Besides, qPCR assays validated that USF1 depletion resulted in obviously decreased GAS6-AS2 levels, while forced expression of USF1 was capable to elevate GAS6-AS2 expression, which indicated that GAS6-AS2 expression was positively correlated with USF1 levels (Figure 2F). To certify that GAS6-AS2 was a transcriptional target of USF1, we respectively cloned serial truncations of GAS6-AS2 promoter (named as site1 to site6, which contained one or more predicted binding sequences) into pGL3 luciferase reporter vectors, and sequentially transfected these constructs into MG63 cells. The luciferase activities were determined and we found that the highest luciferase activities were associated with site1, site4 and site5, but not site2, site3 and site6, which indicated that the predicted binding sequence 2 (B2: -879 to -869: agcacctggca) of GAS6-AS2 promoter was the exact binding sites of USF1 (Figure 2G). For further proving that USF1 was able to bind to B2 sequence of GAS6-AS2 promoter, we next constructed wild-type (WT) or mutant-type (MUT) B2 sequence into pGL3 vector, and performed luciferase activity detection in OS cells after various treatment. We found that enhancing USF1 could dramatically increase the luciferase activities of OS cells transfected with B2 WT luciferase reporter plasmids, while the luciferase activities were not changed in cells co-transfected with pcDNA3.1-USF1 vectors and B2 MUT luciferase reporter plasmids (Figure 2H). Correspondingly, silencing USF1 was able to repress the luciferase activities in cells transfected with B2 WT plasmids. Moreover, to directly demonstrated USF1 was capable to bind to B2 sequences of GAS6-AS2 promoter, we next conducted ChIP assays in MG63 and 143B cells using antibodies against USF1. The results indicated that the complexes precipitated by anti-USF1 antibodies were notably enriched in the GAS6-AS2 promoter DNA fragment, compared with the controls (Figure 2I). In summary, these data validated that USF1 induced the transcription of GAS6-AS2 in OS.

### GAS6-AS2 deficiency inhibited OS cell proliferation and promoted apoptosis *in vitro*

**Figure 3:**
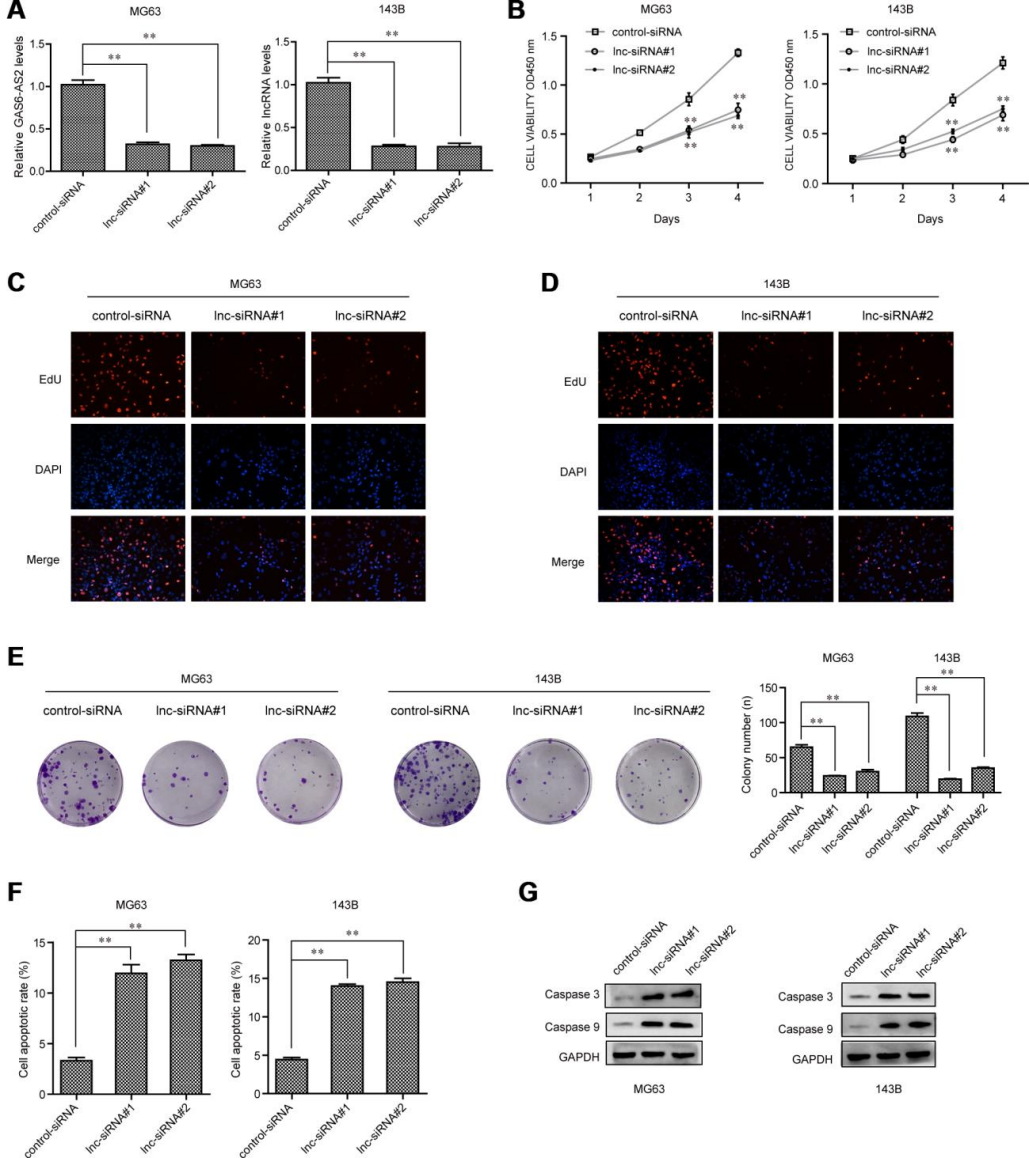
**GAS6-AS2 knockdown impaired the proliferation and promoted cell apoptosis.** (**A**) qPCR detected GAS6-AS2 levels in MG63 and 143B cells after transfection with GAS6-AS2 siRNAs. (**B**) CCK-8 assays. (**C** and **D**) EdU assays. (**E**) Clonogenic assays evaluated the effects of GAS6-AS2 knockdown on cell colony formation capabilities in MG63 and 143B cells. (**F**) Flow cytometry analyses determined the cell apoptosis. (**G**) Western blot assays detected caspase 3/9 protein expression. * P < 0.05, **P < 0.01.

We next sought to investigate the biological functions of GAS6-AS2 in OS cells. Given that GAS6-AS2 was up-regulated in OS cells, we thereby synthesized siRNAs specific target GAS6-AS2 to deplete GAS6-AS2 in OS cells (Figure 3A). CCK-8 assays were then conducted to determine the proliferation capabilities of OS cells (MG63, 143B), confirming the suppressing proliferation of OS cells by GAS6-AS2 knockdown (Figure 3B). Subsequently, EdU assays were also carried out to assess the effects of GAS6-AS2 on cell proliferation. The results revealed that a marked decline in proliferative cell number was observed in OS cells with GAS6-AS2 siRNAs transfection (Figure 3C and 3D). In addition, colony formation assays were conducted to examine the clone formation capacities, demonstrating that the GAS6-AS2 deficiency led to a notable decrease of OS cell clone number (Figure 3E). Furthermore, flow cytometric analyses were also performed to determine whether GAS6-AS2 depletion influenced cell apoptosis. The data verified that repressing GAS6-AS2 expression could markedly accelerate the apoptotic rates of OS cells (Figure 3F). Afterwards, molecular mechanism study using western blot suggested that silencing GAS6-AS2 expression was able to elevate the levels of apoptosis-related molecules, caspase 3 and 9 (Figure 3G). In conclusion, these results indicated that GAS6-AS2 promoted the cellular growth of OS cells, and it might act as a critical regulator in OS development.

### Silence of GAS6-AS2 suppressed OS tumor growth *in vivo*

**Figure 4:**
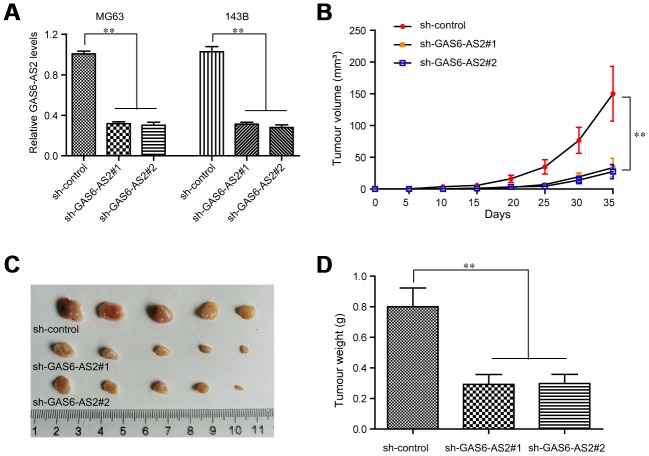
**Knockdown of GAS6-AS2 suppressed OS tumorigenesis *in vivo*.** (**A**) qPCR detected the expression of GAS6-AS2 in OS cells after infecting with GAS6-AS2 shRNA lentivirus. (**B**) The stable lncRNA GAS6-AS2 knockdown MG63 cells were used for *in vivo* tumorigenesis assays. The tumor volumes were recorded every 5 days, and the tumor volume-time curves were shown. (**C**) The photograph and comparison of excised tumor sizes in MG63 cells. (**D**) The tumor weights were analyzed. * P < 0.05, **P < 0.01.

For further clarification of the relationship between GAS6-AS2 and its impact on tumorigenic capabilities *in vivo*, we next constructed GAS6-AS2 shRNA lentivirus vectors and packaged the recombinant lentivirus. The lentivirus which could consistently repress GAS6-AS2 expression (sh-GAS6-AS2#1, sh-GAS6-AS2#2) were respectively applied for infecting OS cells. The knockdown efficiency was determined by qPCR and the data suggested that both sh-GAS6-AS2#1 and sh-GAS6-AS2#2 could markedly reduce the expression of GAS6-AS2 in MG63 and 143B cells (Figure 4A). Afterwards, we injected GAS6-AS2-depleted MG63 cells into the nude mice, and recorded the tumor volumes in each group over time. The data revealed that the tumor volume-time curves were remarkably lower than that of control group, indicating that GAS6-AS2 depletion significantly reduced the volumes of OS tumors (Figure 4B). Then, on the thirty-fifth day, the mice were sacrificed and we found that the tumors in the GAS6-AS2-deficinecy groups were markedly smaller than that of the control group (Figure 4C). Besides, depressing GAS6-AS2 expression dramatically reduced the weights of OS tumors (Figure 4D). Therefore, we demonstrated that depression of GAS6-AS2 inhibited OS cell growth *in vivo*.

### The metastatic potentials of OS cells were impaired by GAS6-AS2 knockdown

**Figure 5:**
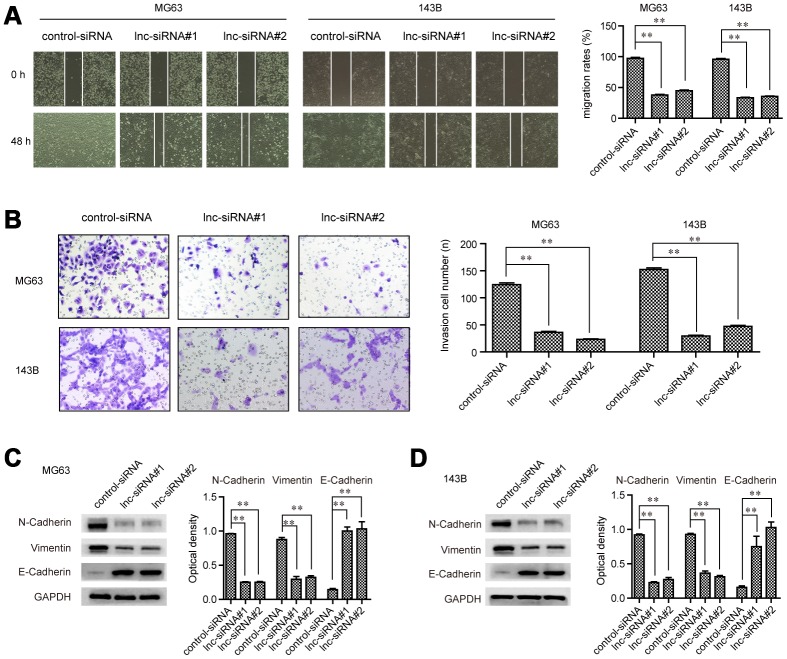
**GAS6-AS2 facilitated the migration and invasion of OS cells.** (**A**) Wound-healing assays. (**B**) Transwell assays. (**C** and **D**) Western blot assays detected protein levels. * P < 0.05, **P < 0.01.

To evaluate whether GAS6-AS2 was involved in modulating OS cell metastatic potentials, wound-healing assays were then conducted. The widths of the wounds in OS cells transfected with GAS6-AS2 siRNAs or control siRNAs were recorded at 0 h and 48 h after scratching. The data proved that depressing the levels of GAS6-AS2 significantly reduced the cell migration, indicating that GAS6-AS2 deficiency decreased OS cell migratory abilities (Figure 5A). Subsequently, transwell assays were carried out to assess the impact of GAS6-AS2 depletion on cellular invasion capacities. The data suggested that repressing GAS6-AS2 expression resulted in a remarkably decreased number of invasive cells, suggesting that GAS6-AS2 silence reduced the invasion abilities of OS cells (Figure 5B). Moreover, the molecular mechanisms by which GAS6-AS2 modulated the metastatic potentials were investigated using western blot analyses. The expression of epithelial-to-mesenchymal (EMT) relevant molecules, N-cadherin, E-cadherin and vimentin, was detected in OS cells after their GAS6-AS2 was knocked down. The data validated that depletion of GAS6-AS2 dramatically reduced N-cadherin and vimentin levels, while GAS6-AS2 silence significantly increased E-cadherin levels (Figure 5C and 5D). Summarily, these data implied that GAS6-AS2 deficiency impaired the migration and invasion abilities of OS cells via regulating EMT molecules.

### MiR-934 was a direct target of GAS6-AS2 in OS

**Figure 6:**
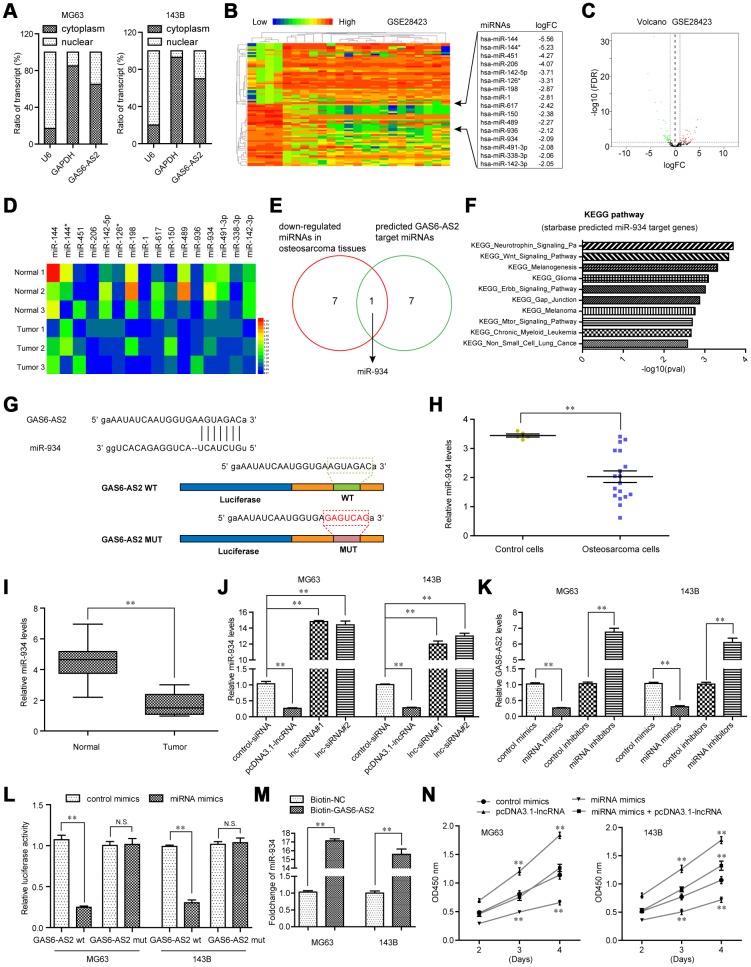
**GAS6-AS2 sponged miR-934 in OS cells.** (**A**) Subcellular fractionation assays were used to determine the subcellular localization of GAS6-AS2 in OS cells. (**B**) Heatmap of differentially expressed miRNAs in GSE28423. (**C**) Volcano map. (**D**) Real-time PCR detected the expression of miRNAs in randomly selected three paired OS tumor specimens and normal tissues. (**E**) Intersection of down-regulated miRNAs in OS and “starbase” algorithm predicted GAS6-AS2 target miRNAs. (**F**) “starbase” algorithm analyzed the KEGG pathway of miR-934 target genes. (**G**) “starbase” algorithm predicted binding sites of GAS6-AS2 and miR-934. (**H**) Relative miR-934 expression in GSE28423 dataset. (**I**) Relative miR-934 expression in 157 OS samples. (**J**) qPCR detected miR-934 levels in MG63 and 143B cells. (**K**) qPCR examined the levels of GAS6-AS2. (**L**) Luciferase activity detection. GAS6-AS2 wt: luciferase reporter containing wild-type binding site of GAS6-AS2 and miR-934. GAS6-AS2 mut: luciferase reporter containing mutant-type binding site of GAS6-AS2 and miR-934. (**M**) RNA-pull down assays. (**N**) CCK-8 assays detected cell proliferation.

Next, we sought to uncover the detail molecular mechanisms by which GAS6-AS2 modulated OS tumorigeneses. Mounting studies had revealed that lncRNAs, especially lncRNAs mainly located in cytoplasm, might regulate the pathogenesis and development of diverse cancer types via competitively binding to specific miRNAs. Hence, we first applied subcellular fractionation assays to determine the subcellular localization of GAS6-AS2 in OS cells. The results suggested that GAS6-AS2 was mainly expressed in cytoplasm, indicating a potential role of GAS6-AS2 in post-transcriptional modulation acting as a ceRNA (Figure 6A). Therefore, we next aimed to discover the target miRNA of GAS6-AS2. To achieve that, we conducted bioinformatics analyses using GSE28423 dataset to preliminarily detect the down-regulated miRNAs in OS, because the target miRNA of GAS6-AS2 might be included in these down-regulated miRNAs (Figure 6B and 6C). We next randomly selected three paired OS tumor samples and normal specimens to determine the levels of the above analyzed down-regulated miRNAs (logFC > -2.0), and the qPCR analysis revealed that seven of these miRNAs (miR-144, miR-142-5p, miR-198, miR-617, miR-489, miR-934, miR-491-3p and miR-142-3p) were down-regulated in OS tumor samples (Figure 6D). In addition, we intersected the above 7 miRNAs with GAS6-AS2 target miRNAs predicted by “starbase” algorithm, and found that only miR-934 was exhibited in both sides (Figure 6E). Indeed, bioinformatics analyses using “starbase” algorithm indicated that the targets of miR-934 were relevant with diverse cancer-related pathways (Figure 6F). Hence, we next focused on miR-934 and attempted to clarify whether miR-934 was a target of GAS6-AS2. The predicted binding site between GAS6-AS2 and miR-934 was presented in Figure 6G. Additionally, miR-934 was also down-regulated in OS cells (analyzing GSE28423 dataset) and 157 OS tumor tissues (Figure 6H and 6I). Besides, real-time PCR analyses confirmed that miR-934 expression was notably abrogated by GAS6-AS2 overexpression, while increased miR-934 expression was observed in cells when GAS6-AS2 was knockdown (Figure 6J). Vice versa, enhancing miR-934 expression could remarkably reduce GAS6-AS2 levels, while miR-934 knockdown elevated the expression of GAS6-AS2 (Figure 6K). These data indicated that GAS6-AS2 expression was negatively correlated with that of miR-934. What’s more, we constructed luciferase reporter plasmids containing the predicted miR-934 binding site (GAS6-AS2 wt) and its matched mutant site (GAS6-AS2 mut). As shown in Figure 6L, co-transfection of miR-934 and GAS6-AS2 wt dramatically reduced the luciferase activities, while co-transfection with miR-934 and GAS6-AS2 mut had no influence on the luciferase activities. To further validate the direct interaction between miR-934 and GAS6-AS2, RNA-pull down assays were carried out. The results suggested that miR-934 was remarkably enriched by biotin-GAS6-AS2 treatment compared with the controls (Figure 6M). These data proved that miR-934 was a direct target of GAS6-AS2 in OS cells. Moreover, functional investigation using CCK-8 assays indicated that overexpression of GAS6-AS2 could significantly restore the suppressing effects of miR-934 on OS cell proliferation (Figure 6N). Taken together, these data demonstrated that miR-934 was a direct target of GAS6-AS2 and GAS6-AS2 was able to regulate OS malignant phenotypes via sponging miR-934.

### BCAT1 promote OS cell proliferation and was a direct target of miR-934 in OS cells

**Figure 7:**
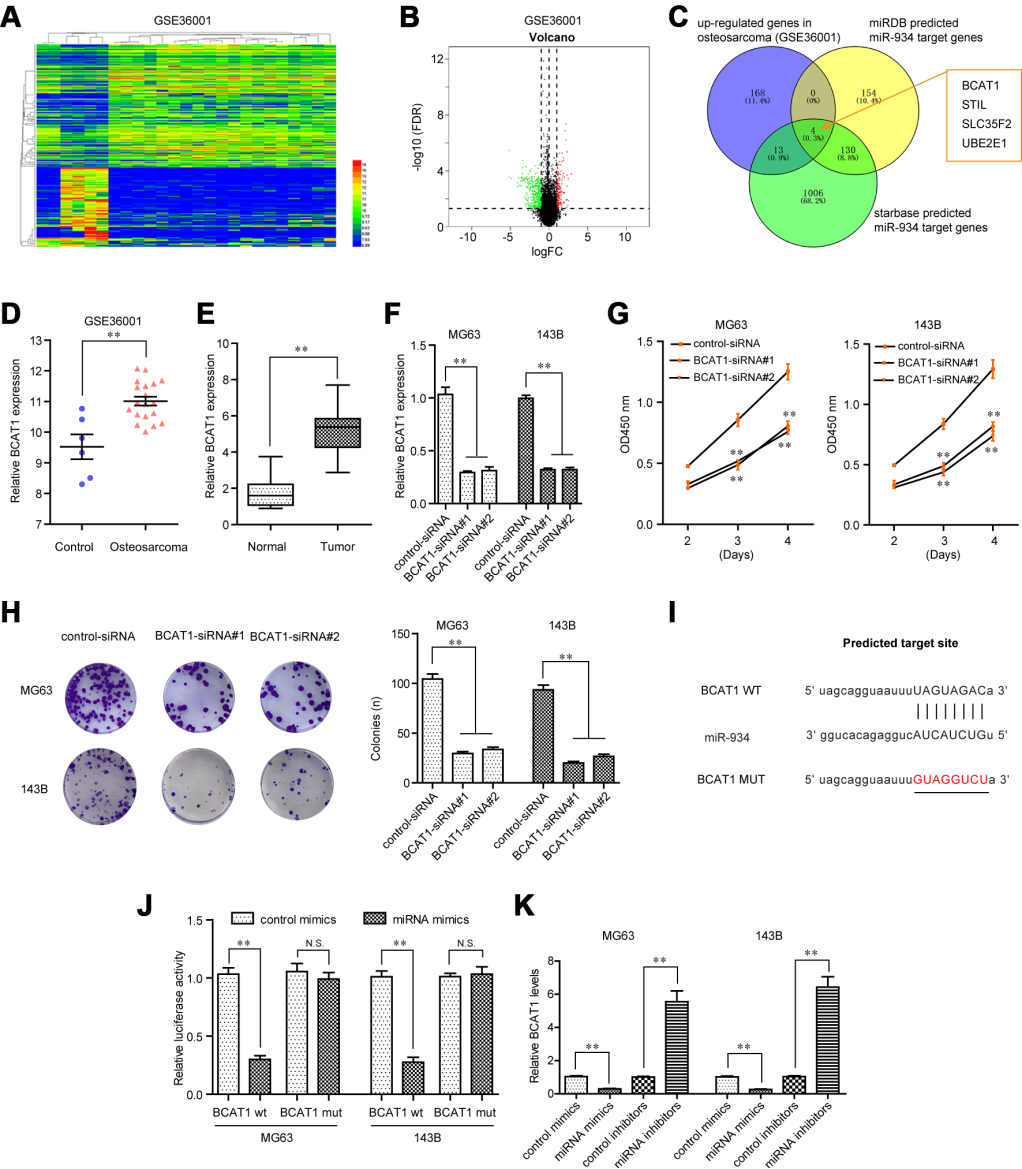
**BCAT1 was targeted by miR-934 in OS cells.** (**A**) Heatmap of differentially expressed genes in GSE36001. (**B**) Volcano map. (**C**) Venn diagram showed the intersection of up-regulated genes in GSE36001 dataset, “miRDB” predicted miR-934 target genes and “starbase” predicted miR-934 target genes. (**D**) Relative BCAT1 expression in GSE36001 dataset. (**E**) Relative BCAT1 expression in 157 OS samples. (**F**) qPCR analyses detected the relative BCAT1 levels. (**G**) CCK-8 assays. (**H**) Clonogenic assays. (**I**) Binding sites between miR-934 and 3’UTR of BCAT1 mRNA. (**J**) Luciferase activity detection. (**K**) Real-time PCR analyses measured relative BCAT1 levels. * P < 0.05, **P < 0.01.

For further elucidation of the molecular mechanism by which GAS6-AS2 exerted its promotive influences on OS malignant phenotypes, we next attempted to discover the possible target genes of miR-934. For that purpose, we first performed bioinformatics analyses using GEO datasets (GSE36001) to obtain up-regulated genes in OS tumor specimens. The heatmap and volcano map of GSE36001 dataset were shown in Figure 7A and 7B. We next intersected the up-regulated genes in OS (GSE36001), “miRDB” predicted miR-934 target genes and “starbase” predicted miR-934 target genes, and found that only four genes (BCAT1, STIL, SLC35F2 and UBE2E1) were commonly exhibited, and BCAT1 ranked first (Figure 7C). Therefore, we next attempted to focus on BCAT1 to study its functions in OS and explore whether BCAT1 was a direct target of miR-934. In fact, bioinformatics analyses using GSE36001 dataset and qPCR assays revealed that BCAT1 was remarkably up-regulated in 157 OS specimens, indicating that BCAT1 might play a role in promoting OS tumor development (Figure 7D and 7E). We thereby synthesized siRNAs specific targeting BCAT1 and subsequently transfected into OS cells. Real-time PCR analyses revealed that BCAT1 siRNAs successfully silenced BCAT1 expression in MG63 and 143B cells (Figure 7F). Functional investigation using CCK-8 assays demonstrated that repressing BCAT1 expression notably attenuated cell growth abilities of OS cells (Figure 7G). Moreover, clonogenic formation analyses confirmed that BCAT1 deficiency dramatically reduced colony formation capacities of OS cells (Figure 7H). Hence, these data suggested that BCAT1 modulated OS development. Next, we sought to investigate whether BCAT1 was a target of miR-934. The “starbase” predicted target sites of miR-934 in 3’UTR of BCAT1 mRNA were presented in Figure 7I. We next cloned luciferase reporters containing the wild-type (wt) predicted miR-934 target site (BCAT1 wt) and its matched mutant site (BCAT1 mut). Afterwards, we transiently transfected with miR-934 mimics into BCAT1 wt- or BCAT1 mut-overexpressing MG63 or 143B cells, and luciferase activity was then detected. The data indicated that forced expression of miR-934 and BCAT1 wt reporters but not BCAT1 mut reporters remarkably reduced luciferase activities (Figure 7J). Additionally, qPCR analyses also indicated that BCAT1 levels were decreased or increased in OS cells transfected with miR-934 mimics or inhibitors, respectively (Figure 7K). Collectively, the above data suggested that BCAT1 was an onco-promoter in OS cells and a direct target of miR-934.

### GAS6-AS2 regulated BCAT1 expression through miR-934 and modulated malignant phenotypes of OS via miR-934/ BCAT1 axis

**Figure 8:**
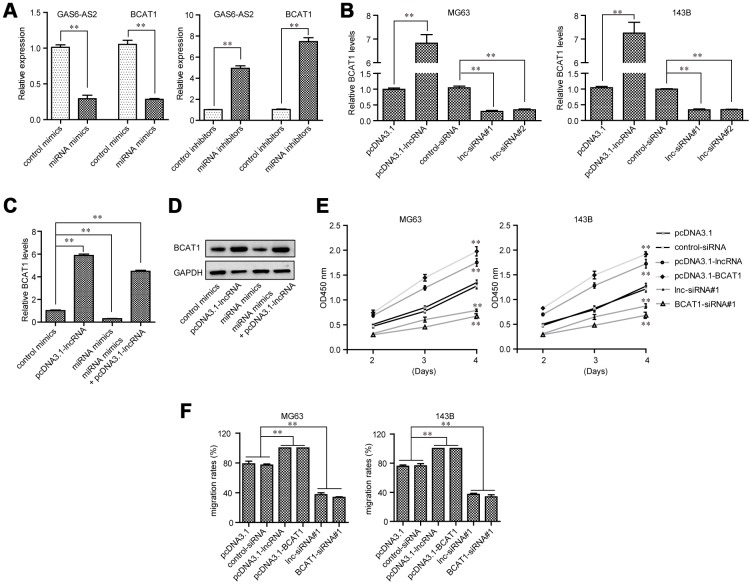
**GAS6-AS2 modulated OS cell malignancies via miR-934/BCAT1 in OS cells.** (**A**) GAS6-AS2 and BCAT1 expressing levels in OS cells were assessed by qPCR analyses. (**B**) Real-time PCR analyzed BCAT1 expressing levels in OS cells after GAS6-AS2 was overexpressed or silenced. (**C**) Real-time PCR determined BCAT1 expressing levels after the upregulation of GAS6-AS2 or overexpression of miR-934. (**D**) Western blot measured BCAT1 proteins. (**E**) CCK-8 assays. (**F**) Wound-healing assays. * P < 0.05, **P < 0.01.

We next sought to assess the expressing relationships among GAS6-AS2, miR-934 and BCAT1 in OS. Real-time PCR analyses demonstrated that ectopic expression of miR-934 obviously depressed the levels of both GAS6-AS2 and BCAT1, while transfection of miR-934 inhibitors notably elevated the expression of GAS6-AS2 and BCAT1, which indicated that miR-934 expression was negatively correlated with GAS6-AS2 and BCAT1 (Figure 8A). Furthermore, qPCR assays elucidated that BCAT1 expression was accelerated by GAS6-AS2 ectopic expression, while BCAT1 expression was depressed in GAS6-AS2-silenced OS cells (Figure 8B). These above data suggested that GAS6-AS2 expression was positively correlated with BCAT1 expression. In addition, we next applied qPCR assays to evaluate the expressing changes of BCAT1 in MG63 cells after their GAS6-AS2 or miR-934 expression was alternated. Although miR-934 could decrease BCAT1 expression, the levels of BCAT1 were remarkably restored by re-enhancing GAS6-AS2 expression (Figure 8C). Similar results were also observed using western blot analyses that forced expression of GAS6-AS2 could reverse the inhibiting influence of miR-934 on BCAT1 protein expression (Figure 8D). Therefore, these above data further demonstrated the regulatory relationships among GAS6-AS2, miR-934 and BCAT1. Besides, CCK-8 assays were conducted to evaluate OS cell proliferation after their GAS6-AS2 or BCAT1 was silenced or ectopic expression. We found that GAS6-AS2 and BCAT1 had similar regulatory effects on OS cell proliferation, that forced GAS6-AS2 or BCAT1 expression markedly promoted cell growth and repressing GAS6-AS2 or BCAT1 expression obviously suppressed cell proliferation (Figure 8E). Moreover, wound-healing assays also indicated that GAS6-AS2 and BCAT1 had similar regulatory impaction on the mobility of OS cells (Figure 8F). Therefore, these results proved that GAS6-AS2 played roles in regulation of OS malignant phenotypes via miR-934/BCAT1 axis.

## DISCUSSION

OS is a contributor to the tumor mortality in children and adolescents. Up to date, the therapeutic strategies in recent years against OS are unsatisfactory and still needed to be further optimized [19]. The identification of novel biomarkers for early detection was necessary for the improved prognosis of OS patients [20]. Recently, the potential of lncRNAs as novel tumor-related biomarkers has attracted growing attentions due to their dysregulation in both tumor tissues and blood of cancer patients and oncogenic roles in controlling tumor proliferation and metastasis [21, 22]. In this study, we identified a novel OS-related lncRNA GAS6-AS2 which may be used as a novel diagnostic and prognostic biomarker for OS patients. For the first time, we reported that GAS6-AS2 was highly expressed in both OS tissues and cell lines. Clinical investigation revealed that overexpression of GAS6-AS2 in OS patients was associated with advanced clinical stage, positively distant metastasis and shorter OS and DFS. More importantly, for further explore the prognostic value of GAS6-AS2 in OS patients, univariate and multivariate analyses were performed and the results confirmed that GAS6-AS2 expression was an independent poor prognostic factor for both 5-year OS and 5-year PFS. Overall, our findings firstly provided clinical evidence that GAS6-AS2 was overexpressed in OS patients and may function as a novel cancer marker.

Previous studies have confirmed that GAS6-AS2 was highly expressed in melanoma and bladder cancer tissues [15, 16]. However, the potential mechanism involved in the upregulation of GAS6-AS2 in the above tumors has not been investigated. In this study, we also showed GAS6-AS2 as an up-regulated lncRNA in OS. In order to explore the mechanism of GAS6-AS2 overexpression in OS, our attention focused on transcription factors which have been reported contributed to lncRNAs dysregulation in human tumors, such as SP1, STAT1 and E2F1 [23–25]. By the use of online software, we identified six transcription factors as potential targets. Among them, USF1 may be the most promising candidate due to its high levels in OS tissues shown in heat map. USF1 contains an HLH-LZ structure and has been confirmed to be involved in the progression of various tumors [26, 27]. In this study, our group provided evidence that overexpression of GAS6-AS2 was induced by USF1, which was affirmed by luciferase reporter assays and ChIP assays. The above experiments revealed that USF1 could bind to the predicted site of the promoter region of CASC11 and activated its transcription. Thus, overexpression of GAS6-AS2 is partly due to the activation of USF1 in OS progression.

Given that the levels of GAS6-AS2 in OS were distinctly up-regulated, we supposed GAS6-AS2 as a contributor in this tumor. Previous study by Wen et al showed that knockdown of GAS6-AS2 suppressed the proliferation and metastasis of melanoma cells, suggesting that GAS6-AS2 as a tumor promoter in this disease [16]. In this study, we also down-regulated the expressions of GAS6-AS2 in MG63 and 143B for the study of GAS6-AS2 function. A series of cells experiments indicated that knockdown of GAS6-AS2 inhibited the proliferation, migration and invasion of OS cells. In addition, we performed Western blot to examine the levels of EMT pathway-related proteins, finding that down-regulation of GAS6-AS2 inhibited the activity of EMT pathway. Overall, our functional investigations suggested GAS6-AS2 as an oncogenic lncRNA in progression of OS.

Competing endogenous RNA (ceRNA) theory, described by Leonardoe et al, attracted huge attention in the study of noncoding RNA [28]. Growing evidence suggested ceRNA as crucial modulators in the posttranscriptional modification, which was involved in the regulation of gene expressions through sponging miRNAs during the progression of various tumors [29, 30]. Thus, our group hypothesized that GAS6-AS2 may serve as a ceRNA engaging in tumorigenesis of OS. We used the bioinformatics databases (Starbase v2.0) to predict the miRNA binding site of GAS6-AS2. In addition, Functional investigation indicated that down-regulation of GAS6-AS2 increased miR-934 expressions. Moreover, the results of the luciferase reporter assays indicated that GAS6-AS2 may be a target of miR-934. Previous studies have reported the distinct dysregulation of miR-934 in several tumors [31, 32]. Further functional assays also provided evidence that miR-934 mediated the cancer-suppressive functions of GAS6-AS2 silence on OS cells. Thus, our findings revealed that GAS6-AS2 may display its tumor-promotive effects by sponging miR-934.

Previous studies have confirmed that miRNAs acted as tumor promoters or oncogenes by targeting various mRNAs [33]. Then, we used TargetScan 7.0 to predict miR-934 target genes. Among these candidate genes, Branched – chain amino acid transaminase 1 (BCAT1) attracted our attention. BCAT1, located in chromosome 12p12.1, has been demonstrated to play a functional role in several biological and pathological processes [34]. Its overexpression was frequently found in many tumors and contributed to the progression of these tumors [35, 36]. However, the expression and function of BCAT1 in OS remains unclear. In this study, we provided evidence that BCAT1 expressions were distinctly up-regulated in OS tissues. Functional exploration suggested that knockdown of BCAT1 inhibited the proliferation of OS cells, indicating it as a tumor promoter in OS. Then, we performed Luciferase reporter assays which confirmed that BCAT1 was a target of miR-934 in OS. Further mechanism assays confirmed that overexpression of miR-934 inhibited the levels of BCAT1, while knockdown of miR-934 had an opposite effect. Finally, the functional assays revealed that GAS6-AS2-miR-934 targets BCAT1 and forms the GAS6-AS2-miR-934-BCAT1 axis to regulate the initiation and progression of OS.

## CONCLUSIONS

We firstly reported that GAS6-AS2 expressions were increased in OS cells and tissues, and was activated by USF1. Our findings also highlighted that GAS6-AS2 may be used as a novel prognostic biomarker for OS patients and acted as a tumor promoter via promoting malignant progression of OS. Mechanically, we revealed a novel GAS6-AS2-miR-934-BCAT1 signaling pathway regulatory network in OS. On the basis of our findings, GAS6-AS2 could be considered as a potential target for the OS treatments in the future.

## MATERIALS AND METHODS

### Tissue samples

One hundred and fifty-seven OS patient specimens were collected following surgery resection in August 2011 to April 2014 from First Affiliated Hospital of Xiamen University. The patient tissue specimens were obtained with written informed consents according to the established protocols approved by the Ethics Committee of First Affiliated Hospital of Xiamen University. Patients did not undergo any chemo- or radio-therapy before surgery. Samples were stored at -80 °C after resection.

### Cell culture

Three OS cells: HOS, U2OS and 143B, were purchased from Yisheng Biological corporation (Jinan, Shandong, China). Other cell lines including MG63, Saos-2 and hFOB1.19 (as control cells), were bought from Degu Biotechnology corporation (Xi’an, Shanxi, China). Cells were allowed to grow in RPMI-1640 media pre-adding with 10% serum. All cell lines were incubated in a 5% CO_2_ incubator at 37 °C.

### SiRNAs, miRNAs, plasmids and transfection

Commercialized-siRNAs (control-siRNA, USF1-siRNA, lnc-siRNA#1, lnc-siRNA#2, BCAT1-siRNA#1, BCAT1-siRNA#2), miRNA mimics or inhibitors, were obtained from Junhong Biotechnology corporation (Jiading, Shanghai, China). Genes or lncRNAs were cloned into pcDNA3.1-basic vectors (such as pcDNA3.1-USF1, pcDNA3.1-GAS6-AS2, pcDNA3.1-BCAT1) to constitutively express them. The constructions were all cloned by Tujie Biological corporation (Binhai, Tianjin, China). For cell transfection, cells (3 × 10^5^ cells per well) were placed into plates (six-well). After the cell confluent reached 70%, siRNAs, miRNAs or plasmids were transfected into the cells using Lipofectamine 2000 reagent kits (Datu, Ningbo, Zhejiang, China).

### Real-time PCR

Total RNAs were purified by using Trizol reagents (Guheng, Jinan, Shandong, China). For mRNAs and lncRNAs examination, cDNA was generated using 1.5 μg RNAs via applying Takara cDNA synthesis kits (Biosheng, Xiamen, Fujian, China). The qPCR analyses using MCE SYBR Green qPCR kits (Dongfu, Qingdao, Shandong, China) were then conducted for the determination of mRNAs or lncRNAs levels. The qPCR conditions were: 94 °C for 30 s, 41 cycles of 94 °C for 5 s, 58 °C for 15 s, and 72 °C for 10 s, followed by the final elongation at 40 °C for 5 min. The mRNAs or lncRNAs expression was evaluated with the 2^−ΔΔCT^ method and the CT values were compared with that for GAPDH. For miRNAs detection, miRNAs were purified by applying Qiangen miRNA extraction kits (Furong, Hefei, Anhui, China). Then, qPCR reactions for miRNAs were conducted using Transgen miRNA qPCR kits (Tengyun, Dalian, Liaoning, China) according to the protocols in the kits. U6 was used as an internal control. The qPCR results were analyzed using the 2^-ΔΔCt^ method. Primers used in this study were shown in Table 4.

**Table 4:** The primer sequences included in this study.

**Name**	**primer sequences (5’–3’)**
GAS6-AS2: forward	AAGGAGGACGCAATACC
GAS6-AS2: reverse	ATCCTGGCTAACACGGT
USF1: forward	CTGCTGTTGTTACTACCCAGG
USF1: reverse	TCTGACTTCGGGGAATAAGGG
miR-934: forward	GCTGTCTACTACTGGAGA
miR-934: reverse	CAGTGCGTGTCGTGGAGT
BCAT1: forward	AGCCCTGCTCTTTGTACTCTT
BCAT1: reverse	CCAGGCTCTTACATACTTGGGA
GAPDH: forward	ACAACTTTGGTATCGTGGAAGG
GAPDH: reverse	GCCATCACGCCACAGTTTC
U6: forward	CTCGCTTCGGCAGCACA
U6: reverse	AACGCTTCACGAATTTGCGT

### Cell proliferation evaluation

Cell proliferation was assessed by Dojindo CCK-8 kits (Zemeng, Xiamen, Fujian, China). In short, cells after treatment were harvested, washed and they (2.5 × 10^3^ cells) were then placed into plates (ninety-six-well). After the cells attaching to the plates, we added 15 μl CCK-8 reagents into each well every twenty-four hours. After incubation for 2.5 h, the absorbance of the plates was recorded at 450 nm wavelength by a microplate reader.

### 5-Ethynyl-2’-deoxyuridine (EdU) assays

The cell proliferation was also determined by EdU assays using Beyotime EdU detection kits (Zhongjin, Changsha, Hunan, China). The cells after treatment were collected and re-placed in the plates (ninety-six-well). On the second day, the cells were incubated with EdU reagents (50 μl; 50 μM) for 2.5 h. Apollo reagents (provided in the kits) were then added into the cells for treatment, followed by being stained with DAPI solution. After treatment with 4% paraformaldehyde (for fixation), EdU-positive cells (red) and DAPI-stained cell nuclei (blue) were observed and photographed by a fluorescence microscope.

### Clonogenic ability detection

Cell clonogenic abilities were determined by colony-formation assays. Briefly, cells after treatment were placed in the plates (six-well) with about 500 cells per well. After two to three weeks incubation, methanol (1.5 ml) with crystal violet (0.2%) was placed into the plates and the plates were incubated at room temperature for 30 min. After washing with PBS, the colonies were photographed by a microscope.

### Cell apoptosis determination

Apoptosis was detected by using the flow cytometry apoptosis detecting kits (AiNuo, Changsha, Hunan, China). The treated OS cells were harvested in PBS buffer (650 μl). Then, the cells were stained by Annexin V-FITC (7 μl) for 15 min, followed by being added with propidium iodide (5 μl). After 20 min, the cells were washed and subjected to flow cytometry analyses.

### *In vivo* tumor formation

Twenty-one BALB/c nude mice (5 weeks; male) were used for xenograft assays. The GAS6-AS2 short hairpin RNA interfering (shRNA) lentivirus was bought from EnFu Biological corporation (Changsha, Hunan, China). The MG63 cells were then cultured to 60-70% cell confluent and infected with GAS6-AS2 shRNA lentivirus. The knockdown efficiency was determined by qPCR analyses as described above. Then, the cells were digested and washed using PBS twice. Afterwards, the cells (1.5 × 107 cells per mouse; 70 μl) were mixed with Matrigel (30 μl), and the mixtures were subcutaneously injected into the right flanks of nude mice. The tumor volumes in each group over time were recorded. The mice were sacrificed on the thirty-fifth day after the tumor cells were injected. Tumor volumes were calculated using the following formula: 1/2 × L × W2, where L represented tumor length and W represented tumor width. The xenograft assays were approved by the Xiamen University Laboratory Animal Care Committees.

### Western blotting

RIPA buffer (Rongfu, Nanchang, Jiangxi, China) was applied for the extraction of total proteins from MG63 or 143B cells after treatment with specific siRNAs. Thereafter, cell lysates (containing 25 μg total proteins) were boiled in loading buffer, followed by being subjected to SDS-PAGE (8-12%). Then, separated proteins were transferred onto PVDF membranes and blocked using 5% BSA solution. Afterwards, the membranes were incubated with primary antibodies overnight at 4 °C. The membranes were then treated using TBST buffer for four times. Subsequently, matched secondary antibodies were used for incubation with the membranes. Finally, ECL kits (Qike, Chengdu, Sichuan, China) were applied for visualizing the protein bands. The anti-caspase 3 and 9 antibodies were bought from Hongjie Biotechnology corporation (Changsha, Hunan, China). Anti-N-cadherin antibody, anti-BCAT1 antibody and anti-GAPDH antibody were bought from Abcam Inc. (Cambridge, MA, USA). Anti-E-cadherin antibody and anti-vimentin antibody were purchased from CST Biotechnology corporations (Danvers, MA, USA).

### Wound-healing assays

Cells were placed in the plates (twenty-four-well) with high density. Then, 1.5 ml media were added into each well. After reaching more than 90% cell confluent, pipettes (200 μl) were used to scratch the cell monolayers and the wounds were generated. The floating and dead cells were washed out using PBS. Cell migration was recorded using a microscope at 0 h and 48 h after the wounds were generated.

### Transwell assays

Cells (1.5 × 10^5^) were placed in 250 μl media (without serum) and subsequently distributed into the upper sides of Millipore transwell insert chambers (8 μl pore size; pre-treated using Matrigel; Yanji, Chengdu, Sichuan, China). As chemo-attractants, 15% FBS containing media were placed into the lower chambers. Twenty-four hours later, cells on the upper sides were removed and cells on the reverse sides (invasive cells) were treated using paraformaldehyde (4%), followed by being stained using crystal violet (0.2%). After washing twice using PBS, the stained cells were imaged by a microscope.

### Subcellular fractionation assays

To clarify the sub-cellular location of GAS6-AS2, the cytoplasmic and nuclear fractions from MG63 and 143B cells were isolated using Life Technologies’ PARIS kits (Boyuan, Wuhan, Hubei, China). Subsequently, RNAs from the isolated cytoplasmic and nuclear fractions were purified and qPCR analyses were applied for determining the expression ratio of specific RNA molecules between the cytoplasmic and nuclear fraction.

### ChIP assays

ChIP assays were carried out according to protocols described by CST SimpleChIP Enzymatic Chromatin IP kits (Heyuan, Nantong, Jiangsu, China). In brief, MG63 or 143B cells were respectively fixed using 1% formaldehyde for 10 min, followed by being quenched for 10 min using glycine (125mM) at room temperature. The cell nuclei were isolated and resuspended in IP buffer according to the kits’ protocols. Then, the DNA was sheared to 200-400 bp fragments by sonication using a Scientz ultrasonic cell disruptor (Ningbo, Zhejiang, China). Thereafter, the chromatins were respectively precipitated using anti-USF1 antibodies or anti-IgG antibodies (control). The purified DNA fragments were finally analyzed by qPCR. The antibodies used in the experiments were bought from Abcam Inc. (Cambridge, MA, USA).

### RNA-pull down

The biotinylated RNAs (biotin-NC, biotin-GAS6-AS2) were obtained from Qiaorui Biological corporation (Dalian, Liaoning, China). In short, the treated MG63 or 143B cells were harvested and added with 550 μl lysis buffer. Then, biotin-GAS6-AS2 (200 pmol) or biotin-NC (200 pmol) was respectively incubated with the above cell lysates (2.5 μg) for 1.5 h at 4 °C. Afterwards, we added Pierce streptavidin-agarose beads (150 μl) into the above mixture. The reactions were then incubated for 35 min, followed by eluting the binding RNAs. Finally, the levels of eluted miR-934 were detected by qPCR analyses.

### Luciferase activity detection

The site1-6 sequences of GAS6-AS2 promoter were respectively cloned into pGL3-basic luciferase reporter vectors and named as site1, site2, site3, site4, site5 and site6. In addition, the wild-type (WT) or mutant-type (MUT) sequence containing “JASPAR” predicted binding position 2 (B2) was subcloned into pGL3 vector, and the luciferase vector was named as B2 WT or B2 MUT, respectively. Besides, the “starbase” predicted GAS6-AS2 wild-type (wt) or corresponding mutant-type (mut) sequence of the miR-934 target site was also constructed into pGL3-basic vector, and named as: GAS6-AS2 wt and GAS6-AS2 mut, respectively. Similarly, luciferase reporter plasmid containing “starbase” predicted binding site between miR-934 and 3’UTR of BCAT1 was named as BCAT1 wt, and its matched mutant-type luciferase reporter plasmid was named as BCAT1 mut. All the constructs were generated by Saixun Biological corporation (Chengdu, Sichuan, China). For luciferase activity detection, cells were placed into ninety-six-well plates (4000 cells per well). After the cells were attachment, they were transiently transfected with a mixture of indicted reporter plasmids or miRNA mimics. Forty-eight hours later, the luciferase activity was detected by using Promega luciferase reporter assay kits (Kejia, Hangzhou, Zhejiang, China).

### Statistical analyses

The student's t-test or one-way ANOVA was applied for statistical analyses using 19.0 statistical software (IBM, Armonk, New York, USA). Kaplan-Meier curve plot and log rank test were employed to analyze overall and disease-free survival rates. Univariate and multivariate analyses of the prognostic factors were performed with Cox regression model. P value less than 0.05 was considered statistically significant.

### Ethics approval

The present study was authorized by the Ethics Committee of the Xiang’an Hospital of Xiamen University, School of Medicine, Xiamen University. All procedures performed in studies were in accordance with the ethical standards. All patients and volunteers were anonymous and have provided written informed consent.
